# The cumulative risk of lung cancer among current, ex- and never-smokers in European men

**DOI:** 10.1038/sj.bjc.6602078

**Published:** 2004-08-03

**Authors:** A Crispo, P Brennan, K-H Jöckel, A Schaffrath-Rosario, H-E Wichmann, F Nyberg, L Simonato, F Merletti, F Forastiere, P Boffetta, S Darby

**Affiliations:** 1 Epidemiology Unit, National Cancer Institute, 80131 Naples, Italy; 2 International Agency for Research on Cancer, 69008 Lyon, France; 3 Institute for Medical Informatics, Biometry and Epidemiology, 45122 Essen, Germany; 4 Institute of Epidemiology, GSF National Research Center, 85764 Neuherberg, Germany; 5 Institute of Environmental Medicine, Karolinska Institute, 17177 Stockholm, Sweden; 6 Department of Environmental Medicine and Public Health, University of Padova, Italy; 7 Unit of Cancer Epidemiology, CeRMS and Centre for Oncologic Prevention, University of Turin, 10125 Turin, Italy; 8 Department of Epidemiology, Rome E Health Authority, Rome, Italy; 9 Clinical Trials Service Unit (CTSU), Radcliffe Infirmary, Oxford OX2 6HE, UK

**Keywords:** lung cancer, cigarette smoking

## Abstract

Recent analyses based on UK data indicate that people who stop smoking, even well into middle age, avoid most of their subsequent risk of lung cancer. We investigated whether similar absolute risks of lung cancer in men are found in other European countries with different smoking patterns and at different stages of their lung cancer epidemic. Using data for men from a multicentre case–control study of lung cancer in the UK, Germany, Italy and Sweden, and including 6523 lung cancer cases and 9468 controls, we combined odds ratio estimates with estimates of national lung cancer incidence rates to calculate the cumulative risk of lung cancer among men by age 75. Lung cancer cumulative risks by age 75 among continuing smokers were similar for the UK, Germany and Italy at 15.7, 14.3 and 13.8% respectively, whereas the cumulative risk among Swedish male smokers was 6.6%. The proportion of the risk of lung cancer avoided by quitting smoking before the age of 40 was comparable between the four countries, at 80% in Italy and 91% in the UK, Germany and Sweden. Similarly, the proportion of the excess risk avoided by quitting before the age of 50 ranged from 57% in Italy to 69% in Germany. Our results support the important conclusion that for long-term smokers, giving up smoking in middle age avoids most of the subsequent risk of lung cancer, and that lung cancer mortality in European men over the next three decades will be determined by the extent to which current smokers can successfully quit smoking.

Smoking of tobacco products, and in particular of cigarettes, is responsible for most cases of lung cancer in Europe and North America, with an attributable proportion among men in the order of 90% (Peto *et al*, 1992; Mannino *et al*, 2001; Simonato *et al*, 2001; Jemal *et al*, 2003). The association between lung cancer and cigarette smoking is strongly related to duration of smoking, and to a lesser extent the amount of tobacco smoked per day, the type of cigarette and other characteristics such as inhalation patterns (Lubin *et al*, 1984; Boffetta *et al*, 1999). Measuring precisely the absolute risk of lung cancer for various patterns of smoking (e.g. heavy smokers, long-term ex-smokers) typically requires information on large cohorts with long-term follow-up information. A more efficient, although rarely used, approach (Cornfield, 1951) is to combine measures of odds ratio (OR) from case–control studies with relevant incidence or mortality data. A recent example was based on a case–control study of lung cancer in southwest England including 667 male cases and 2108 male controls (Peto *et al*, 2000). By incorporating national UK mortality rates for lung cancer in males, the cumulative risk of death from lung cancer by age 75 among current smokers was estimated at 16%, rising to 24% for current smokers of at least 25 cigarettes per day. The benefit of quitting smoking was demonstrated with cumulative risks of lung cancer of 10, 6, 3 and 2% for men who stopped smoking at ages 60, 50, 40 and 30, respectively. It is therefore apparent that smokers who quit, even well into middle age, avoid a large proportion of their subsequent risk of lung cancer. Using a larger multicentre case–control study of lung cancer in Europe, which included the above UK data (Simonato *et al*, 2001), we investigated whether the absolute risks of lung cancer in men are similar in other European countries with different smoking patterns and in different stages of the lung cancer epidemic.

## MATERIALS AND METHODS

The analysis was based on a multicentre case–control study of lung cancer among men conducted from 1985 to 1994 in the UK, Germany, Italy and Sweden (Jöckel *et al*, 1998; Nyberg *et al*, 2000; Peto *et al*, 2000; Kreienbrock *et al*, 2001; Fortes *et al*, 2003; Kreuzer *et al*, 2003; Richiardi *et al*, 2004). The UK data formed the basis of the previous absolute risk analysis (Peto *et al*, 2000). Three centres were in Germany, three in Italy and one each in Sweden and the UK (Table 1

**Table 1:** Distribution of the study subjects by centre and case–control status

**Centre**	**Reference^a^**	**Case/control**	**Region**	**Source of cases**	**Source of controls**
UK	Peto *et al* (2000)	667/2108	Southwest England	Residents in Devon and Cornwall	Population registers and patients from hospitals of cases
					
Germany					Population registers and random digit dialling
Total		3464/3501			
Germany I	Jöckel *et al* (1998)	805/799	(I) Bremen, Frankfurt	(I) Residents in Bremen, Frankfurt metropolitan areas	
Germany II	Kreienbrock *et al* (2001)	1850/1796	(II) Parts of Northrhine-Westfalia, Rhineland-Palatine, Saarland, Eastern Bavaria	(II) Residents in parts of Northrhine-Westfalia, Rhineland-Palatine, Saarland, Eastern Bavaria	
Germany III	Kreuzer *et al* (2003)	809/906	(III) Thuringia and Saxony	(III) Residents in Thuringia and Saxony	
					
Italy					Population registers and patients from hospital of cases
Total		1377/1535			
Italy I	Richiardi *et al* (2004)	538/684	(I) Turin	(I) Residents in Turin	
Italy II	Richiardi *et al* (2004)	510/582	(II) Veneto region	(II) Five areas of Veneto region	
Italy III	Fortes *et al* (2003)	329/269	(III) Rome	(III) Patients from one hospital in Rome	
					
Sweden	Nyberg *et al* (2000)	1015/2324	Stockholm county	Residents in Stockholm county	Population registers

a Number of subjects included in pooled analysis do not correspond exactly with numbers of subjects in original publications.

). Cases were incident patients with lung cancer aged below 75 years and, for Germany, Italy and Sweden, histologically or cytologically confirmed. Controls were a random sample of the underlying populations selected from local population registers, with the exception of Centre III in Italy, in which controls were selected from patients admitted to the same hospital as the cases for a disease unrelated to smoking. In the UK, a separate hospital control group was also recruited using criteria similar to those in the Italian study. In Sweden, besides the population controls in Stockholm, a second group of mortality-matched controls was selected from among those who died in the same year as the cases but from a nontobacco-related cause of death. For both Sweden and the UK, the distribution of the smoking habits in the two control groups was similar andEpidemiology, CeRMS and Authority, Rome, Italy; 9Clinical the results are presented here with the two groups combined.

All subjects who had regularly smoked at least one cigarette per day for at least 1 year (6 months in Germany) were considered current or ex-smokers. Current smokers were those who still smoked at interview or had quit less than 2 years previously (1 year in the UK), while ex-smokers were those who had quit 2 or 3 years previously. When information on different time periods was available for current smokers, the most recent number of cigarettes smoked was used. Information on other tobacco smoking products was also included after weighting for the amount of tobacco (one pipe=one cigar=1.5 cigarillos=3 cigarettes). Smokers of only cigars or pipes were excluded from the analysis in the UK data set.

Country-specific relative risks of lung cancer were estimated by means of OR with 95% confidence intervals using unconditional logistic regression (Breslow and Day, 1980) and adjusting for age. Smoking categories were introduced as dummy variables, and ORs were estimated for each smoking category compared to never-smokers. Overall results for Germany and Italy were calculated by pooling the data from the three centres in each country and adjusting by centre.

The absolute risks were estimated by combining the male age-specific cancer incidence rate in each country with OR for the different smoking categories and the age-specific prevalence of the different smoking habits among study controls (see Appendix A1 for further details). Our assumption was that within each age group the smoking distribution of each national population was represented by our control distribution. Finally, we calculated the cumulative rate (*C*) for the different categories of smoker by adding age-specific absolute rates (in 5-year age groups), and then the cumulative risk by age 75 using the standard formula (1−exp(−5*C*/10^5^)) (Breslow and Day, 1987). The cumulative risk may be interpreted as the probability that an individual will develop lung cancer before the age of 75 in the absence of competing causes of death.

National lung cancer incidence rates for the period 1988–1992 were used for the UK and Sweden (Parkin *et al*, 2003). As national lung cancer incidence rates are not available for Germany and Italy, we estimated these by using the age-specific national lung cancer mortality data corrected by survival rates to obtain an adjusted incidence rate:


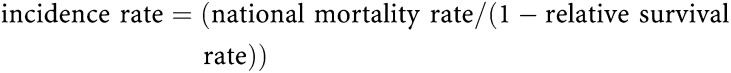


Age-specific lung cancer mortality rates were averaged over the period 1988–1994, covering the period when most subjects were recruited. Country-specific lung cancer relative survival data (adjusted for competing causes of death) were obtained from the EUROCARE study (Janssen-Heijnen *et al*, 1998) covering the period 1985–1994.

95% confidence intervals for cumulative risks were calculated using floating absolute risks to obtain the variance of the logarithm of OR Var(log* r*) (Easton *et al*, 1991) and subsequently incorporating a Taylor series expansion. The method of floating absolute risks leads to confidence intervals that are approximately independent and are therefore readily interpreted (Plummer, 2004). Sampling variations in smoking prevalence estimates are not included in the calculation of the confidence intervals, resulting in intervals that may be somewhat too narrow. Further details are given in Appendix A1.

## RESULTS

A total of 6523 cases and 9468 controls have been included in the analysis. The ORs associated with cigarette smoking are presented in Table 2

**Table 2:** Odds ratios and cumulative risks of lung cancer by age 75, for never-, ex- and current cigarette smokers, among men, by country

**Country**	**Case/control**	**OR^a^**	**Standard^a^ 95% CI**	**Floating^b^ 95% CI**	**Cumulative risk^c^ (95% CI)**
UK^d^					
Nonsmoker	3/400	1.0		0.3–3.1	0.2 (0.01–0.4)
Ex-smoker	285/1106	34.4	10.9–107.5	30.2–39.1	5.7 (5.0–6.4)
Current smoker	322/453	94.8	30.2–297.8	82.2–109.3	15.7 (14.4–17.0)
					
Germany					
Nonsmoker	63/763	1.0		0.8–1.3	0.6 (0.4–0.7)
Ex-smoker	1059/1699	7.5	5.7–9.8	7.0–8.1	4.2 (4.0–4.5)
Current smoker	2342/1039	27.3	20.9–35.6	25.4–29.4	14.3 (13.6–15.0)
					
Italy					
Nonsmoker	18/277	1.0		0.6–1.6	0.6 (0.3–0.9)
Ex-smoker	520/716	11.2	6.8–18.2	10.0–12.5	6.5 (5.9–7.0)
Current smoker	833/539	23.7	14.6–38.7	21.3–26.5	13.8 (12.8–14.8)
					
Sweden					
Nonsmoker	36/706	1.0		0.7–1.4	0.4 (0.2–0.5)
Ex-smoker	263/813	6.3	4.4–9.1	5.5–7.3	2.3 (2.0–2.6)
Current smoker	716/805	17.4	12.3–24.7	15.3–19.3	6.6 (6.2–7.0)

a Odds ratios and 95% confidence intervals calculated by logistic regression analyses, adjusted for age and centre.

b 95% confidence intervals are based on floating variance.

c Cumulative risk expressed as percentage; 95% confidence intervals are based on floating variance.

d UK estimates of cumulative risk differ slightly from those previously reported (Peto *et al*, 2000) due to use of incidence instead of mortality data. The OR for ex-smokers and current smokers in UK are also anomalously high by chance, due to the fact that the number of nonsmokers was unusually low by chance. This anomaly will have a minimal effect on the UK cumulative risks for current and ex-smokers. See text for an explanation.

, those for current and ex-smokers being higher in the UK than in the other countries, although this is based on very few (three) lifelong nonsmoking cases in the UK. Cumulative risks for never-smokers are relatively consistent between the four centres, ranging from 0.2% in the UK to 0.6% in Italy and Germany. Cumulative risks among ex–smokers were 4.2% in Germany, 5.7% in the UK and 6.5% in Italy, while in current smokers they were 14.3% in Germany, 15.7% in the UK and 13.8% in Italy. In Sweden, however, these cumulative risks were somewhat lower at 2.3% for ex-smokers and 6.6% for current smokers.

In Table 3

**Table 3:** Odds ratios and cumulative risks of lung cancer by age 75, by age at quitting smoking, among men, by country

**Centres**	**Case/control**	**OR^a^**	**Standard^a^ 95% CI**	**Floating^b^ 95% CI**	**Cumulative risk^c^ (95% CI)**
UK^d^					
Nonsmoker	3/400	1.0		0.3–3.1	0.2 (0.01–0.4)
<30 years	6/122	6.6	1.6–26.6	2.9–14.9	1.1 (0.3–1.9)
40 years	12/193	8.3	2.3–29.7	4.6–14.9	2.6 (1.3–3.9)
50 years	44/246	23.8	7.3–77.6	17.3–32.9	5.6 (4.1–7.0)
60 years	223/545	54.6	17.3–171.7	46.7–63.8	11.1 (9.4–12.8)
Current smoker	322/453	94.8	30.2–297.8	82.2–109.3	15.7 (14.4–17.0)
					
Germany					
Nonsmoker	63/763	1.0		0.8–1.3	0.6 (0.4–0.7)
<30 years	27/294	1.03	0.6–1.6	0.7–1.5	0.6 (0.3–0.8)
40 years	52/426	2.8	1.9–3.9	2.2–3.5	1.8 (1.4–2.1)
50 years	169/498	6.8	5.0–9.1	5.9–7.9	4.9 (4.4–5.4)
60 years	804/481	15.0	11.2–20.1	13.0–17.3	10.8 (10.0–11.6)
Current smoker	2342/1039	27.3	20.9–35.6	25.4–29.4	14.3 (13.6–15.0)
					
Italy					
Nonsmoker	18/277	1.0		0.6–1.6	0.6 (0.3–0.9)
<30 years	34/103	5.6	2.9–10.6	3.6–8.6	3.4 (2.0–4.9)
40 years	61/131	4.9	2.7–9.0	3.4–7.2	3.3 (2.1–4.4)
50 years	130/268	8.9	5.2–15.1	7.0–11.3	6.3 (5.1–7.5)
60 years	295/214	12.7	7.6–21.2	10.5–15.4	10.2 (9.1–11.2)
Current smoker	833/539	23.7	14.6–38.7	21.3–26.5	13.8 (12.8–14.8)
					
Sweden					
Nonsmoker	36/706	1.0		0.7–1.4	0.4 (0.2–0.5)
<30 years	4/71	1.1	0.4–3.2	0.4–3.0	0.4 (0.01–0.8)
40 years	14/158	1.7	0.9–3.3	1.0–3.0	0.8 (0.4–1.1)
50 years	46/215	4.2	2.6–6.6	3.1–5.8	2.1 (1.6–2.6)
60 years	199/369	7.2	4.7–10.9	5.7–9.3	4.2 (3.6–4.8)
Current smoker	716/805	17.4	12.3–24.7	15.3–19.3	6.6 (6.2–7.0)

a Odds ratio and 95% confidence intervals calculated by logistic regression analyses, adjusted for age and centre.

b 95% confidence intervals are based on floating variance.

c Cumulative risk expressed as percentage; 95% confidence intervals are based on floating variance.

d See footnote to Table 2 concerning the anomalously high odds ratios for the UK study.

, cumulative risks among ex-smokers are presented after stratifying for age at quitting. For the UK, the cumulative risk of lung cancer by age 75 was 11.1, 5.6 and 2.6% among men who stopped at around 60, 50 and 40 years of age, respectively. For Germany and Italy, the cumulative risks of lung cancer by age 75 among ex-smokers were similar to those obtained for the UK. For Sweden, the cumulative risk by age 75 was 4.2, 2.1 and 0.8% for those who stopped at around 60, 50 and 40 years of age. These results are presented in further detail in Figure 1

**Figure 1:**
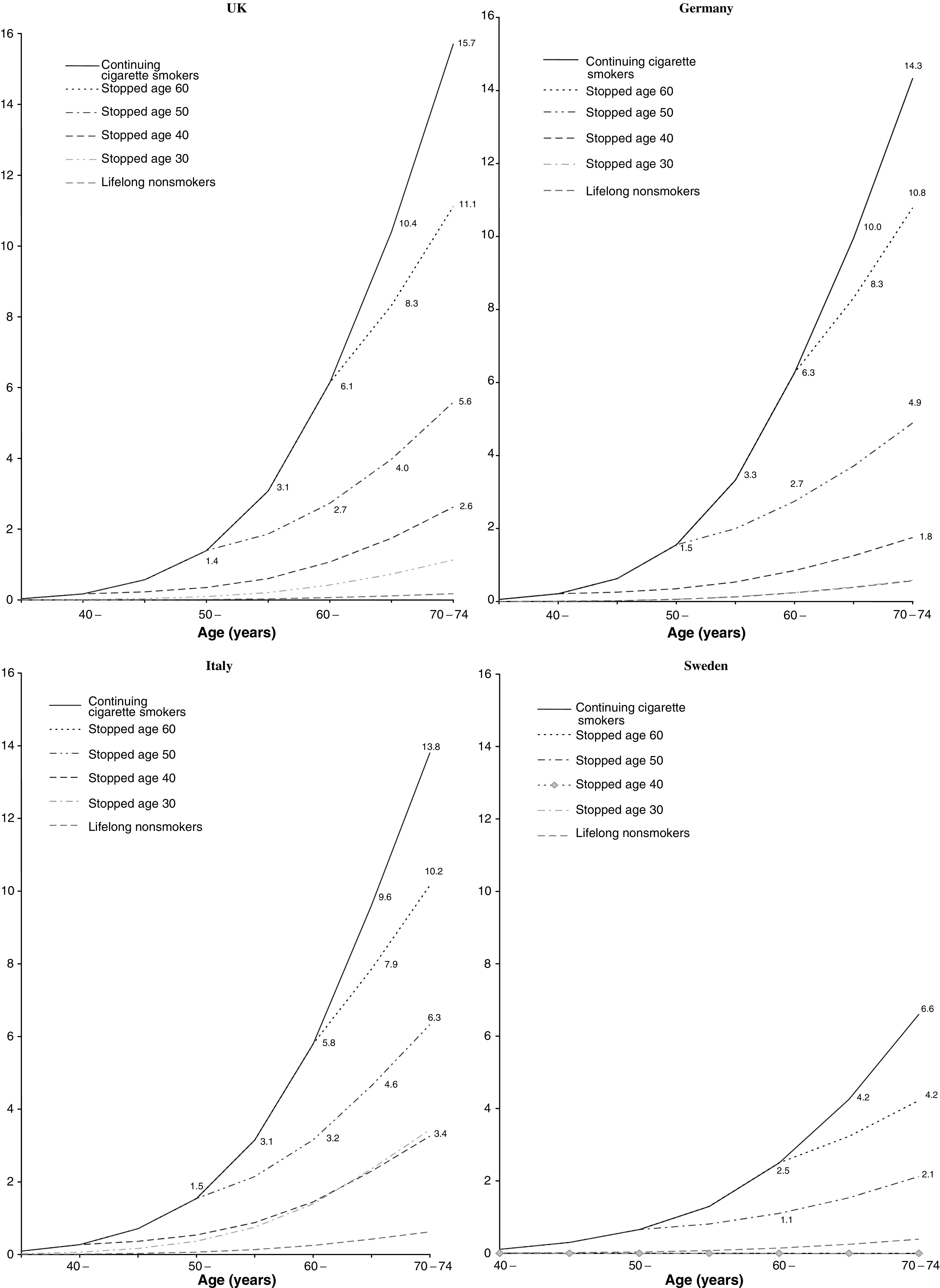
Effects of stopping smoking at various ages on the cumulative risk (%) of death from lung cancer up to age 75 at incidence rate for men in Europe.

, which includes the age-specific cumulative risk in each category of smoking.

The cumulative risks for current smokers according to the number of cigarettes currently smoked per day are presented in Table 4

**Table 4:** Odds ratios and cumulative risks of lung cancer by age 75, for current cigarette smokers stratified by amount smoked per day (most recent estimate), among men, by country

**Centres**	**Case/control**	**OR^a^**	**Standard^a^ 95% CI**	**Floating^b^ 95% CI**	**Cumulative risk^c^ (95% CI)**
UK^d^					
Nonsmoker		1.0		0.3–3.1	0.2 (0.01–0.4)
<5 day−1	20/44	60.6	17.3–212.0	35.7–102.8	10.4 (5.8–14.8)
5–14 day−1	108/179	80.9	25.2–256.7	63.3–102.1	13.1 (11.2–16.0)
15–24 day^−1^	126/169	99.4	31.2–316.7	78.9–125.2	16.5 (13.7–19.2)
25+ day^−1^	68/61	148.6	45.3–487.0	105.2–210.0	23.6 (17.5–29.3)
					
Germany					
Nonsmoker	63/763	1.0		0.8–1.3	0.6 (0.4–0.7)
<5 day^−1^	28/91	4.5	2.4–8.4	2.5–8.0	2.5(2.1–3.0)
5–14 day^−1^	576/412	17.6	13.1–23.6	15.3–20.3	9.5 (9.0–10.1)
15–24 day^−1^	1171/385	33.6	25.3–44.5	29.9–37.8	17.4 (16.6–18.2)
25+ day^−1^	567/147	52.2	37.7–73.2	42.8–63.6	25.7 (24.3–27.0)
					
Italy					
Nonsmoker	18/277	1.0		0.6–1.6	0.6 (0.3–0.9)
<5 day^−1^	10/32	3.0	1.1–8.1	1.2–7.1	1.8 (1.4–2.3)
5–14 day^−1^	206/202	13.9	8.2–22.4	11.3–17.1	8.3 (7.6–9.0)
15–24 day^−1^	395/208	26.8	16.1–44.5	22.5–31.9	15.4 (14.4–16.4)
25+ day^−1^	222/97	35.9	21.0–61.4	28.0–46.0	20.1 (18.6–21.5)
					
Sweden					
Nonsmoker	36/706	1.0		0.7–1.4	0.4 (0.2–0.5)
<5 day^−1^	14/14	19.6	8.7–44.2	9.3–41.1	7.0 (4.8–9.2)
5–14 day^−1^	193/346	10.9	7.5–15.9	9.2–13.0	4.0 (3.6–4.3)
15–24 day^−1^	320/325	19.3	13.3–27.9	16.5–22.5	6.9 (6.5–7.4)
25+ day^−1^	189/79	36.2	23.5–55.8	27.6–47.7	12.6 (11.3–13.9)

a Odds ratios and 95% confidence intervals calculated by logistic regression analyses, adjusted for age and centre.

b 95% confidence intervals are based on floating variance.

c Cumulative risk expressed as percentage; 95% confidence intervals are based on floating variance.

d The cumulative risks for smokers of <5 day^−1^ are anomalously high in UK. Of these cases, 93% had smoked over 15 day^−1^ in the past, and several had smoked over 30.

. Among heavy smokers (25+ per day), risks were similar in the UK, Germany and Italy at 23.6, 25.7 and 20.1% respectively, while in Sweden they were much lower at 12.6%. The pattern among moderate smokers (15–24 per day) was much the same as for heavy smokers, although risks were somewhat lower. For light smokers (<5 per day), risks were similar in Germany and Italy at 2.5 and 1.8, although were higher in the UK and Sweden. This is probably due to the larger amount of cigarettes these ‘light’ smokers had smoked in the past. For example, over 90% of the lung cancer cases in the UK who were classified as light smokers had previously smoked over 15 cigarettes per day.

## DISCUSSION

The primary aim of the analysis was to determine whether the absolute risks of developing lung cancer for various groups of smokers in different European countries are similar to those previously reported for the UK (Peto *et al*, 2000). A high degree of consistency was observed between results for the UK, Germany and Italy. In all three countries, the cumulative risk of developing lung cancer by age 75 for continuing smokers was around 14–16%, or about one in seven, and the risk for continuing heavy smokers was around 20–25%, or around one in four to one in five. Given that some of these heavy smokers may have been light smokers previously, it is possible that the true cumulative risk for those who have been heavy smokers throughout their adult lives may be even higher. Finally, the relationship between lung cancer risk and quitting smoking was very similar in the UK, Germany and Italy. Overall, the proportion of the excess absolute risk of lung cancer by age 75 compared to never-smokers that was avoided by quitting smoking at age 40 was 85, 91 and 80% in the UK, Germany and Italy respectively. Similarly, the proportion of the excess lung cancer risk avoided by quitting at age 50 was 65, 69 and 57%, respectively. A substantially lower cumulative risk was observed in Sweden, with the lifetime risk of current smokers up to age 75 being approximately half that identified in the other three countries. However, the proportion of the excess lung cancer risk avoided by quitting was similar to the other three countries, with 94 and 73% of the excess risk being avoided by those who quit before age 40 and 50, respectively.

The heterogeneity of the results from Sweden as compared to the other three countries raises the possibility of bias in our results. However, there is no obvious reason why the cumulative risk in the Swedish study was seriously underestimated. The study recruited a series of incident cases and two control groups, which were subsequently combined: one a group of population controls selected among the Stockholm population alive at the end of the year of recruitment among the cases, and the second a group of controls frequency-matched to cases on vital status at the end of the study period. Individuals who had died of a tobacco-related cause were not included in this second control group (Gustavsson *et al*, 2000). The Swedish study was restricted to Stockholm county, whereas cumulative risks were calculated based on national incidence rates. We therefore repeated the analysis of the Swedish data using incidence data from Stockholm county. The resulting cumulative risks by age 75 in Stockholm County were 0.4, 2.4 and 6.5% for never-, ex- and current smokers respectively, which are similar to the figures obtained using national figures.

A further possible explanation is that Swedish men may have started smoking at a later age than in other countries. We therefore repeated the calculation of cumulative risk after stratifying for age at onset of smoking. Among continuing smokers who started smoking before the age of 16, the cumulative risk of lung cancer by age 75 was 16.5% in the UK, 20.7% in Germany, 21.7% in Italy and 11.5% in Sweden. Results from such stratified analyses are unstable due to smaller numbers, although they do not indicate that the lower cumulative risks observed in Sweden are fully explained by a later age of onset of smoking. Other possible explanations for the low cumulative lung cancer risk among Swedish men may be different inhalation patterns, smoking of a higher proportion of low-tar cigarettes, and simultaneous use of smokeless tobacco products resulting in a reduced intensity of smoking (Foulds *et al*, 2003), although no information was collected on these aspects.

The age-standardised mortality rate for lung cancer among Swedish males is substantially lower than in other European countries, at 22.6 per 100 000 for the year 2000, while current mortality rates in the UK (48.6), Germany (46.2) and Italy (52.6) are more typical of other European countries (Ferlay *et al*, 2001). The difference between Sweden and the other countries cannot be explained by differences in consumption. In 1950, consumption of manufactured cigarettes per adult per day was 1.8 in Italy and West Germany, 2.2 in Sweden and 6.0 in the UK (Forey *et al*, 2002). Separate figures are not available for men and women, although it is likely that the vast majority of cigarettes were smoked by men in all these countries. In Italy and Sweden, consumption subsequently rose steadily after 1950, reaching 6.3 and 4.5 respectively by 1985. In West Germany and the UK, consumption increased from 1950 and peaked in the early 1970s at 7.3 and 8.8 respectively, with subsequent declines to 6.4 and 5.9 respectively by 1985. In all these countries, the proportion of cigarettes that were smoked by women increased during the period 1950–1985, although to a lesser extent in Italy than elsewhere.

The anomalously high ORs for current and ex-smokers for the UK are attributable to the fact that only three nonsmoking lung cancer cases were found in this study, corresponding to a cumulative risk by age 75 of 0.2%. The majority of the evidence concerning lung cancer risks in lifelong nonsmokers in the UK indicates that they are similar to those seen in the American Cancer Society (ACS) prospective study, corresponding to a cumulative risk by age 75 of around 0.4% (Peto *et al*, 1992), suggesting that the low number of nonsmokers observed in the study was a chance finding. In a previous analysis of data from this study in which the ACS data were used for nonsmokers, the ratio of risks in current smokers compared with nonsmokers was estimated to be 100 : 3, that is, about 33.3 (Peto *et al*, 2000). The same argument would also reduce the ratio of risks in ex-smokers to nonsmokers by a factor of about three. This would suggest that the ORs for current and ex-smokers in the UK are in fact quite compatible with those observed for Germany and Italy.

The anomalously high ORs for the UK have only a minimal effect on the cumulative risks. The reason for this is that the sum of the cumulative risks for all three categories of smokers combined is constrained to be equal to the total cumulative risk as determined by the absolute rate of lung cancer. As the estimated cumulative risk among lifelong nonsmokers in the UK is probably about 0.2% too low, the sum of the cumulative risks for the other two categories is probably about 0.2% too high. The cumulative risks for ex-smokers and current smokers in the UK as given in Table 2 are therefore probably each too high by about 0.1%.

The present study was restricted to lung cancer, although, considering its implications, it is noteworthy that a comparably large benefit of stopping smoking was found for all-cause mortality in the prospective study among British doctors (Doll *et al*, 1994). In a recent review of smoking-related mortality in developed countries (Peto *et al*, 2003), it has been estimated that during the year 2000 in the 15 countries, then members of the European Union, there were 172 000 smoking-attributed male deaths at ages 35–69, comprising 29% of all male deaths in this age range, while for all ages the corresponding figures were 398 000 and 22%. In the 10 new member states of the European Union, the proportion of deaths attributable to smoking is even higher. It is estimated that, in 2000, 37% of all male deaths at ages 35–69 and 28% of all male deaths were attributable to smoking, corresponding to 73 000 deaths at ages 35–39 and 113 000 at all ages. The estimates of cumulative risk obtained in the present study reinforce the conclusion that, for people who have been smoking for many years, giving up smoking in middle age means avoiding most of the subsequent risk of developing lung cancer or of dying from a tobacco-related disease. This result has major public health implications. Tobacco-related mortality in Europe over the next three decades will be determined by the extent to which current smokers can be persuaded to quit smoking.

## Appendix A1

The cumulative risk was calculated using methods similar to those described by Peto *et al* (2000). The following box summarises the main measures used to obtain the cumulative risk:

**Table:**

*r*_*i*_ is the relative risk in the *i*_*i*_th smoking category	(1)
where the smoking categories are: 1=never-smokers, 2=ex-smokers (subdivided in some analyses by age at which they quit smoking (<30, 30− 40−, 50−, 60+), 3=current smokers subdivided in some analyses by number of cigarettes per day (<5, 5−14, 15−24, 25+)	
*p*_*ij*_ is the percentage of the controls in the *j*th age group and the *i*th smoking category	(2)
where the age categories are 35–39, 40−44, 45−49, 50−54, 55−59, 60−64, 65−69 and 70−74 years	
*S*_*j*_=*r*_*1*_*p*_*1j*_+⋯+*r*_*i*_*p*_*ij*_	(3)
*h*_*j*_ is the age-specific incidence rate	(4)
*f*_*j*_=*h*_*j*_/(*S*_1_+⋯+*S*_*j*_)	(5)
*a*_*ij*_ is the absolute rate in the (*i,j*)th cell, and *a*_*ij*_=(*f*_*j*_*r*_*i*_)	(6)
*C*_*i*_ is the cumulative rate, and *C*_*i*_=∑_*j*_*R*_*j*_*a*_*ij*_	(7)
where *R*_*j*_ is the width of the *j*th age category in years	
cumulative risk (%)=100(1−exp(−*C*_*i*_))	(8)

Briefly, to estimate the cumulative risk, first we need to calculate the relative risks in the various smoking categories (1). In our study, the relative risks of lung cancer were estimated by means of OR using unconditional logistic regression, and lifelong nonsmokers formed the reference category.

The second step was to calculate the percentage of controls in each smoking category and for each age group (2) using the assumption that within each country the smoking distribution of the population was represented by the smoking distribution observed among the study controls.

The third step was the estimation of common factors combining the relative risk (1) for the different smoking categories with the age-specific prevalence of such smoking habits among study controls (2), thus obtaining the quantities denoted by (3).

Combining the age-specific cancer incidence rates of each country (4) with the common factors (3), we obtained the proportions given in (5). Multiplying these proportions with the relative risks for the different smoking categories produced the age-specific absolute risks in the different smoking categories (6).

Next we calculated the cumulative rates (7) for the different categories of smoker by adding age-specific absolute rates, and then finally the cumulative risks by age 75 were estimated using the standard formula (8). The cumulative risk may be interpreted as the probability that an individual will develop lung cancer before age 75 in the absence of competing causes of death.
